# The next generation of physician-researchers: undergraduate medical students’ and residents’ attitudes, challenges, and approaches towards addressing them

**DOI:** 10.1186/s12909-024-06166-8

**Published:** 2024-11-14

**Authors:** Behnaz Mokhtari, Reza Badalzadeh, Saeideh Ghaffarifar

**Affiliations:** 1 Medical Education Research Center, Tabriz University of Medical Sciences, Tabriz, Iran; 2 Molecular Medicine Research Center, Tabriz University of Medical Sciences, Tabriz, Iran; 3 Department of Physiology, Faculty of Medicine, Tabriz University of Medical Sciences, Tabriz, Iran; 4 Department of Medical Education, Education Development Center, Tabriz University of Medical Sciences, Tabriz, Iran

**Keywords:** Curricula, Education, Medical students, Physician, Research activities, Residency

## Abstract

**Background:**

Undergraduate medical education and residency training are critical periods for conducting research. Medical diagnoses and therapies are direct results of successful research efforts that have advanced several scientific fields. This review highlights the importance of incorporating scientific research training into the curricula of undergraduate medical education and residency programs.

**Methods:**

We utilized key databases such as PubMed, Web of Science, and Google Scholar to conduct a narrative review of English-language articles published between 2002 and 2024. Ultimately, we selected 49 studies that examined the attitudes of undergraduate medical students and residents toward research, the common challenges they encounter while participating in research activities, and the strategies that support and encourage their involvement, all in alignment with our study objectives and keywords. In addition, we identified several relevant themes, including the value of research experience in shaping well-rounded medical professionals; the integrity of research practices in residency selection, which addresses concerns such as the arms race and misrepresentation, as well as the balance between quality and quantity; striking a balance between research and clinical training while promoting equity and inclusion; and effective programs and mentorship strategies to enhance research engagement.

**Results:**

Translating the positive attitudes of undergraduate medical students and residents into improved knowledge and practice necessitates the development of structured mentoring programs and advanced training systems. Institutions must provide the necessary tools, guidance, and support to overcome research barriers. This will help establish an environment in undergraduate medical education and residency training that values research, facilitates access to it, and integrates it into the curriculum.

**Conclusions:**

To address the critical shortage of physician-researchers and improve evidence-based medical practice, it is crucial for medical schools to focus on research education and create avenues for student involvement. By tackling research challenges and implementing supportive strategies, these efforts empower the next generation of physician-researchers to embrace research, contribute to medical progress, and uphold the highest standards of patient care.

## Background

Recently, there has been a significant shift from experience-based practices to evidence-based practices in both medicine and education. Evidence-based medical practice relies on research, which is fundamental for translating new knowledge and technological advancements into effective tools for preventing and treating diseases. The exponential growth of research projects can be attributed to the rapid progress in developing new drugs, therapies, and devices. Major changes in medical education and practice have resulted from the publication of research studies, particularly landmark trials. Clinical trials provide clinicians with an opportunity to collect controlled observations through a scientific and objective approach, empowering them to make informed decisions about the most effective therapy for each patient based on the most reliable data available [1, 2].

Research training is a crucial aspect of medical education, serving several important purposes. One of the primary benefits is the cultivation of a pipeline of physician-researchers. Through their dual role, physician-researchers effectively bridge the gap between the research and practice communities, contributing to the improvement of both medical services and research endeavors. Their efforts facilitate the development of clinically relevant research and promote the widespread adoption of evidence-based treatments in standard clinical practices. Engaging in research allows physician-researchers to acquire valuable insights regarding the latest treatments and technologies. These insights can be directly applied to their clinical practice, resulting in improved patient care and outcomes. Furthermore, physician-researchers can assist in identifying research questions that align with the interests of physicians and have distinct potential for enhancing patient care [3–5]. Research experience also helps learners become better consumers of medical literature. By understanding research methodologies and data interpretation, physicians can critically appraise studies and discern their applicability to individual patients or communities, which is crucial for evidence-based practice [6–8].

In many regions, research experience is a requirement for the accreditation of medical training programs, ensuring that all trainees receive a comprehensive education that includes exposure to research. Involvement in research can enhance the competitiveness of medical students, residents, and fellows when applying for the next stages of their careers. It demonstrates a commitment to academic excellence and can distinguish candidates in a competitive field. The value placed on research can vary significantly among countries, specialties, and career paths. Some countries may emphasize research more in their accreditation processes, while others may focus more on clinical skills. Similarly, certain specialties may prioritize research experience more than others, making it important to understand these variabilities for a global perspective on the topic [9–11].

Medical residency training is a period of rigorous education and a platform for medical graduates to experience significant personal and professional development. During this time, residents acquire the ability to apply theoretical knowledge in practical clinical settings and develop the skills necessary for patient care. However, residency training is not just about clinical rotations and patient care; it also involves research [12]. Involvement in academic research and scholarly activities during residency training is crucial for medical residents to stay updated with the latest advancements, develop a deep understanding of their field, and prepare for successful careers. Engaging in research can also make residents more competitive for fellowships and fellows more competitive for academic jobs. This is particularly important given the variability in the emphasis on research among different countries, specialties, and career paths [13–15]. Surgical training, in particular, presents significant challenges and demands, including uncertainties, increasing responsibilities, sleep deprivation, and personal sacrifices. Therefore, research should not be perceived as an additional burden alongside clinical duties. Instead, it is advisable to integrate research into the daily routines of surgical residents to enhance their research endeavors. Nonetheless, this integration raises concerns regarding surgical caseloads, especially in countries with duty hour constraints [16, 17].

Attitudes and barriers toward research are pivotal factors that influence the success of research endeavors. Negative attitudes toward research hamper the learning process and are correlated with inadequate research performance [18]. Moreover, engaging in research during undergraduate medical education and residency training is challenging due to personal limitations, lack of interest, insufficient knowledge of research methodology, time constraints, heavy clinical workloads, absence of mentors, and limited resources. It is crucial to address the research challenges faced by undergraduate medical students and residents and to employ various strategies to enhance the research culture [19–21]. The objective of this review was to outline the attitudes of undergraduate medical students and residents toward research, the common challenges they experience when participating in research activities, and the approaches that promote their research participation.

## Methods

We conducted a narrative review of English-language articles published between January 2002 and January 2024 through literature searches of core databases, including PubMed, Web of Science, and Google Scholar. Based on our study objectives and identified keywords, which included Medical Subject Headings (MeSH) terms, we ultimately selected 49 studies that explored the attitudes of undergraduate medical students and residents toward research, the common challenges they encounter in engaging with research activities, and the strategies that facilitate and encourage their participation. To achieve this, we developed a set of keywords: ‘medical students OR medical residents AND research OR attitudes OR challenges/barriers/obstacles AND approaches/solutions.’ While our primary focus was on the attitudes, barriers, and solutions related to medical research involvement, we also examined several other pertinent themes within the selected articles. These themes were identified based on their relevance to the broader context of medical education and research engagement. Additional areas of investigation included the value of research experience in shaping well-rounded medical professionals; the integrity of research practices in residency selection, addressing issues such as the arms race, misrepresentation, and the balance between quality and quantity; the challenge of balancing research and clinical training while promoting equity and inclusion; and effective programs and mentorship strategies to enhance research engagement. It should be noted that as the review progressed, themes were categorized, discussed, revised, and reviewed by all authors in an ongoing cycle until consensus and data saturation were achieved. At this point, the information obtained from additional articles no longer provided new insights or significantly contributed to our study. Consequently, we decided to exclude these articles from our review to maintain focus and ensure that our study was based on the most relevant and impactful findings. This approach allowed us to concentrate on articles that offered unique perspectives and valuable contributions to our understanding of the topic, thereby enhancing the overall quality of our narrative review. Ultimately, after several consultations among all authors, the categories developed into six distinct themes. This review was carried out in compliance with the ethical guidelines established by the Ethics Board of Tabriz University of Medical Sciences (Ethics Approval Number: IR.TBZMED.REC.1400.959).

## Results

### Beyond academia: the value of research experience in shaping well-rounded medical professionals

Research participation in medical education has garnered increasing attention as a key factor influencing the academic development of medical students. While it is acknowledged that not all medical students aspire to become academics or pursue research-intensive careers, the benefits of engaging in research extend far beyond the act of publication itself. Engaging in research can significantly enhance students’ skills, including critical thinking, problem-solving, data analysis, and scientific communication—essential competencies in the medical field. The demand to promote evidence-based medical practice and bench-to-bedside translational research, led by physician-researchers, has emerged as the primary motivator for incorporating research into medical education [22, 23]. As a result, there is now a heightened focus on making research a fundamental part of modern medical education, accompanied by an increasing sense of urgency [24–26].

Studies have demonstrated that participation in medical student journals is a strong predictor of both short- and long-term academic success. For instance, one study revealed that even limited participation in research activities can foster skills that contribute to better academic performance. This suggests that early exposure to research can lay a foundation for future academic endeavors, regardless of whether students ultimately choose a research-focused career [27]. Moreover, early research experiences are pivotal in shaping the development of physician-scientists. These experiences provide students with valuable skills such as analytical thinking, teamwork, and effective communication, which are applicable across various medical disciplines. Therefore, students who do not pursue research careers can still gain valuable competencies through their involvement in research activities [28]. Recognizing the diverse aspirations of medical students, it is essential to offer robust educational opportunities that cater to all learners. This includes providing comprehensive educational programs that emphasize clinical skills, patient care, and other essential competencies for those who may not pursue research. A balanced approach ensures that all students receive a well-rounded education that adequately prepares them for their future roles in medicine, fostering a generation of well-equipped healthcare professionals [24–26].

### Reevaluating research practices and integrity in residency selection: addressing the arms race, misrepresentation, and quality over quantity

The current residency selection process has created an “arms race” where applicants feel pressured to engage in research activities to improve their chances of securing a position. This competitive landscape fosters a focus on research output over meaningful engagement, often leading to the misrepresentation of scholarly achievements and raising critical questions about the quality of research produced and the integrity of application processes. Consequently, there is a pressing need to shift the focus of trainee research toward advancing science and patient care rather than perpetuating this arms race, which contributes to research pollution. The system should discourage poor research practices, including poorly designed studies, incorrect analyses, selective interpretation, and fraud. Eliminating the emphasis on publication quantity and limiting the number of research outputs listed on applications could shift the focus toward quality. This approach would maintain high standards while fostering a more meaningful research culture in residency selection, prioritizing fewer, more impactful studies conducted for the right reasons [29].

To further understand the implications of this “arms race” in research productivity, a study explored U.S. residency program directors’ views on medical student research, emphasizing its crucial role in disseminating research findings and developing transferable skills. It also explored the potential impact of the 2022 transition of the United States Medical Licensing Examination (USMLE) Step 1 to a pass/fail format on the significance of research. This change has introduced uncertainty about how research and other application components will affect interview and ranking decisions. Active participation in research not only enhances self-efficacy but also fosters genuine interest and scholarly abilities among medical students. Program directors value meaningful research engagement, recognizing essential attributes such as curiosity and critical thinking. The shift to a pass/fail Step 1 exam has amplified the importance of research in residency applications, making it imperative for students to engage in research to enhance their competitiveness. As residency application criteria evolve, research emerges as a crucial differentiator, highlighting the necessity for students to actively participate in research activities to bolster their residency match prospects [30]. The study by Radulovich et al. at a community medical school examined how this transition affects medical students’ perceptions of research. Findings indicated that while the shift may alleviate some exam pressure, students increasingly recognize the need to strengthen other application components, particularly research experience. To address these challenges, medical schools should integrate research opportunities with community initiatives, ensuring that students engage in meaningful research that benefits both their academic profiles and the communities they serve. Facilitating mentor matching and establishing dedicated research programs aligned with community health needs can enhance the quality of research conducted. Furthermore, implementing intensive research mentorship is vital for guiding students through the evolving residency application landscape while maintaining a commitment to community engagement. As the emphasis on research continues to grow, community-based medical schools must prioritize both research integrity and community service. Future research should focus on balancing these critical components to prepare students as both competent researchers and dedicated community advocates [31].

Building on the concerns regarding research integrity and the pressures faced by medical students, misrepresentation of scholarly achievements emerges as a significant issue. Misrepresentation of scholarly achievements is a significant concern, particularly in the medical field, where professional accomplishments are vulnerable to embellishment. Kistka et al. addressed the critical issue of publication misrepresentation among residency applicants and underscored the need for enhanced education and supervision of medical students to ensure they are well-informed about the importance of accurate reporting and adhere to rigorous standards in evaluating research contributions. Institutions should revise application guidelines to ensure clearer reporting and verification of research contributions, distinguish between different types of research outputs—such as peer-reviewed publications, conference presentations, and posters—and consider the significance of authorship position and journal quality in assessing authenticity [32]. These issues underscore the importance of not only addressing misrepresentation but also emphasizing the quality of research outputs in the evaluation process. In line with this, the quality of research is paramount, and an increase in quantity does not inherently lead to better outcomes. A study by Karri et al. indicated that the rising competitiveness in dermatology has indeed led to an increase in reported research output among applicants. However, this trend is not accompanied by a proportional increase in peer-reviewed publications, raising concerns about the reliance on non-indexed research outputs, such as abstracts and presentations. These outputs may inflate perceived research productivity without necessarily reflecting quality. Therefore, the findings emphasize the need for residency programs to adopt a more rigorous and comprehensive method of evaluating research outputs. It is crucial to prioritize quality peer-reviewed publications over non-indexed items to ensure that applicants are genuinely showcasing their research capabilities [33].

### Balancing research and clinical training while promoting equity and inclusion

Increased research in medical education may inadvertently reduce clinical exposure, especially in orthopedic surgery, where Cerasani et al. highlighted a looming physician shortage in underserved areas by 2025. The study recommends that residency programs value candidates committed to community health through research and global health initiatives. Therefore, medical schools must balance research activities with clinical training to ensure a well-rounded education. Additionally, accommodating more research may require realistic adjustments in faculty time, considering patient needs, faculty well-being, and reimbursement structures. While integrating research into the curriculum is crucial for advancing medical knowledge, it should not compromise essential clinical training. Future research should investigate how curricular innovations can effectively prepare students for careers in both academic and clinical settings, particularly in underserved areas [34]. Furthermore, as medical schools navigate the integration of research into their curricula, it is imperative to address disparities in research opportunities and outcomes among students. A study revealed disparities in publication rates among medical students based on sex and racial/ethnic identity, highlighting the need for interventions to promote equity and inclusion. It underscores the importance of addressing systemic biases and providing equitable research opportunities to all students to foster a diverse biomedical research workforce. Addressing these disparities, particularly among women and underrepresented in medicine (URIM) students, is crucial for creating an inclusive environment in medical education and research [35].

### Attitudes of undergraduate medical students and residents towards participation in research

The attitudes of undergraduate medical students and residents towards research are essential factors that determine the quantity and quality of research conducted during their education and training. Attitudes toward research among undergraduate medical students and residents vary widely. While some view research as a valuable opportunity to contribute to the medical knowledge base and enhance their academic careers, others consider it a burden that diverts their attention from clinical responsibilities [36–38]. There are several ways in which a positive attitude towards research can impact the quality of research work conducted by undergraduate medical students and residents. A positive attitude towards research increases their motivation to engage in research activities, learn new skills and techniques, and dedicate the necessary effort to produce high-quality work. Moreover, when undergraduate medical students and residents have a positive attitude toward research, it enhances collaboration among themselves and with their mentors, thereby promoting more productive and high-quality research projects [9, 20, 39]. Since cultivating positive attitudes toward scientific research is a vital part of modern undergraduate medical education and residency training curricula, we have collected data from several valuable studies to gain additional insights into the attitudes of undergraduate medical students and residents toward engaging in research activities.

#### Study 1

An investigation was conducted to evaluate the perspectives of anesthesiology residents and program directors on research training within anesthesiology residency programs in Canada. The results showed that while 81% of Canadian anesthesiology residency programs require research participation, only 41% of residents support making research mandatory. Furthermore, 75% of residents expressed a preference for engaging in other academic activities, such as pursuing postgraduate programs in education or acquiring proficiency in transesophageal echocardiography, rather than conducting a research project throughout their residency [40].

#### Study 2

An anonymous, cross-sectional self-report questionnaire administered to second- and fourth-year medical students at three medical schools in Ontario revealed notable differences between the two groups in their attitudes toward mandatory research as part of critical inquiry and scholarly activities in the undergraduate curriculum. There was a significant increase in research participation from the second to the fourth year of medical school. Specifically, 49% of second-year students reported minimal or no involvement in research, compared to only 14% of fourth-year students. While most fourth-year medical students participated in research activities to some extent, only 36% felt confident in their ability to develop a strong understanding of research methodology upon graduation [41].

#### Study 3

A study conducted at Jordan University of Science and Technology examined medical students’ insights on the importance of a research thesis for earning a Doctor of Medicine degree. The findings showed that students have a strong, positive attitude toward conducting research and believe that completing a research thesis should be a requirement for graduation. This study recommended that medical schools make curriculum modifications to incorporate research methodology and provide more support and resources to students interested in conducting research [11].

#### Study 4

The findings of an online survey conducted among obstetrics and gynecology residents in Canada revealed a significant disparity in their attitudes toward scholarly research activity during their residency. Many residents reported that their participation in research was solely due to the requirements set by their program, and some expressed that their training environment did not actively encourage research. Specifically, among the respondents, 94% cited their program’s requirement as one of the motivators behind their involvement in research, 72% reported being engaged in research to advance their careers, and 27% disagreed with the statement that personal interest was a driving force behind their participation [42].

#### Study 5

A study aimed at examining the attitudes of residents from three major departments—Internal Medicine, Pediatrics, and Anesthesiology—at King Chulalongkorn Memorial Hospital in Thailand toward conducting research revealed that 52.4% of respondents expressed a desire to engage in scholarly activities to enhance their research experience. Some residents showed interest in publishing their work in medical journals (12.6%) or presenting at international conferences (4.5%). However, 39.8% of residents demonstrated a lack of motivation to engage in research. Most residents agreed that participating in research motivated them to engage in self-study and enhanced their understanding of research methodology principles. Despite their belief that research could be complex, time-consuming, and tedious, the majority agreed that involvement in research activities had a positive impact on their inquiry-based learning and improved their comprehension of research methodology [43].

#### Study 6

According to the findings of a study investigating the attitudes of residents from an established Accreditation Council for Graduate Medical Education-accredited internal medicine residency training program in Singapore toward research, participation in research activities was observed among one-third of the residents. Over 50% of the residents who actively participated in research dedicated 10–50% of their time to activities directly related to their research. This finding is significant, considering that residents typically dedicate a substantial portion of their training time to clinical responsibilities. The ability of this group of residents to actively participate in research, despite various barriers and a lack of dedicated time for research, is intriguing. The study also documented that the level of research activity and career interest among residents is influenced by specific beliefs; therefore, the lower-than-expected participation rate observed in the surveyed group may indicate a lack of strong belief in the intrinsic value of research. Active research participation was found to be associated with the completion of a postgraduate examination, which potentially provided additional time for research activities. Evidence supports the notion that implementing work-hour restrictions has granted residents the opportunity to pursue academic interests, resulting in a noticeable increase in the number of research publications by residents. However, despite adhering to the restriction of 80 work hours per week, the research involvement rate within this training program continued to be low [44].

#### Study 7

According to the findings of a questionnaire distributed by a national task force of Canadian plastic surgery trainees to all 13 plastic surgery programs in Canada, over 70% of residents expressed interest in pursuing research during their residency, and 74% of the programs have incorporated a research prerequisite into their curriculum. Residents demonstrated several key motivators for conducting research, which included the following: achieving admission into a fellowship program (78%), enhancing the quality of patient care (71%), advancing their academic careers (67%), and improving their proficiency in evidence-based surgical and clinical practices (64%). A small percentage of residents (5%) reported a lack of motivation to engage in research. Interestingly, no correlation was found between university research ranking and the scholarly output of residents or their perceptions of research obstacles [45].

#### Study 8

A survey conducted by Mansi et al. evaluated the attitudes of otolaryngology residents and program directors toward research during residency training. According to the survey findings, research is an essential element of otolaryngology residency training. All residents surveyed reported having some research experience before starting their residency. Furthermore, 33% of the residents stated their intention to pursue an academic career after completing their residency. To reorganize residency programs in a way that promotes research, several potential solutions have been proposed. These include allocating a dedicated block of four months for protected research time, providing residents with support staff and mentors to assist them in their research endeavors, and offering educational opportunities in research methodology and biostatistics to address certain weaknesses among residents [39].

#### Study 9

The results of a study focused on examining the perceived attitudes toward research activities among interns and residents in Rwanda demonstrated that 98% of participants expressed a strong interest in pursuing future research endeavors. Positive attitudes toward research were observed among both interns and residents. Notably, interns showed a greater preference for conducting retrospective research compared to pediatric residents. Only 27% of the students reported submitting their research work for publication [46].

#### Study 10

Based on the evaluation of attitudes and practices related to research among undergraduate medical students at Iuliu Hatieganu University of Medicine and Pharmacy in Cluj-Napoca, Romania, over 60% of third- and fifth-year students expressed interest and willingness to engage in research during their medical studies. Additionally, more than two-thirds of the students indicated a desire to continue pursuing research after completing their education. Notably, among fifth-year students, males demonstrated a higher level of research engagement compared to females [47].

#### Study 11

The percentage of clinical scientists in radiology has historically been low, underscoring the need to increase the pipeline of trainees interested in research. A study examined the perspectives of radiology program directors on MD-PhD trainees, residents’ research productivity, and the allocation of dedicated research time. Program directors generally viewed MD-PhD residents positively, particularly those engaged in research, although they perceived their clinical performance as inferior to that of non-PhD residents. Research productivity during residency was similar between MD-PhD and non-PhD residents, suggesting that it is driven by individual motivation rather than prior training. The study emphasizes the importance of the Medical Scientist Training Program (MSTP) in providing integrated research and medical training while also highlighting challenges faced by MD-PhD graduates in radiology, such as limited research time and income disparities between academic and private practice careers [48].

### Research challenges faced by undergraduate medical students and residents and proposed solutions

To facitilate the participation of undergraduates in the research field, it is necessary to gain a comprehensive understanding of their capabilities and the potential barriers they may encounter. Identifying research barriers can support program directors and policymakers in developing effective interventions that promote research participation among undergraduate medical students and residents. This, in turn, has the potential to enhance patient care and improve outcomes [49, 50]. Several studies have examined research barriers faced by undergraduate medical students and residents and have got very interesting findings. By reviewing key studies, we aimed to highlight effective methods for engaging undergraduate medical students and residents in high-quality research experiences, underscore the critical role of research in medical education and residency training, and emphasize the need for developing physician-scientists (Table 1).

**Table 1:** Overview of research challenges and proposed solutions for undergraduate medical students and residents

No	Study Population	Place and Date of Work	Challenges	Solutions	Reference
1	Internal medicine residents	American College of Physicians-American Society of Internal Medicine annual session, 2002	1. Lack of sufficient time 2. Inadequate research skills 3. Absence of a research curriculum	1. Introduce and implement a research curriculum that explicitly teaches research skills 2. Provide protected time for residents to engage in scholarly work 3. Establish funding opportunities to support resident research endeavors 4. Promote mentorship and collaboration between residents and faculty members 5. Make research a mandatory element of residency training	[51]
2	Medical students	Thirteen institutions in six Brazilian states, 2006–2007	1. Lack of institutional incentives 2. Inadequate infrastructure 3. Limited mentorship time from professors	1. Develop structured research programs 2. Implement policies providing incentives for students and faculty 3. Allocate resources for training opportunities 4. Revise curricula to ensure students acquire essential research skills 5. Collaborate with public and private sectors to improve research facilities	[52]
3	Medical students	Kasturba Medical College, Mangalore, 2007	1. Shortage of time 2. Lack of financial or academic incentives 3. General disinterest in pursuing research as a career	1. Enhance research training 2. Develop support systems 3. Address perceptions of research careers 4. Encourage student participation 5. Consider gender dynamics	[53]
4	Pediatric residents	United States, 2012	1. Lack of time 2. Lack of faculty expertise 3. Lack of funding	1. Enhance resident training through structured research programs 2. Understand the variability in available resources, and address barriers and low satisfaction rates among program directors 3. Implement a scholarly activity requirement and provide faculty mentorship 4. Standardize guidelines to ensure consistency and effectiveness in research training	[54]
5	Medical students	College of Medicine, Taibah University, Madinah, Saudi Arabia, 2014	1. Inadequate facilities for research 2. Lack of interest from faculty or mentors 3. Unavailability of samples or patients	1. Enhance research training 2. Promote gender inclusivity 3. Encourage faculty engagement	[55]
6	Final-year medical students who completed two years of research training	University of Medical Sciences and Technology in Sudan, which has provided research training for 17 years, 2015	1. Insufficient funding 2. Limited time 3. Demands of the curriculum	1. Adopt a small group learning model to enhance students’ research training and ensure effective supervision for group research projects 2. Revise the curriculum and investigate student theses to identify deficiencies in supervision, skills, and knowledge 3. Conduct further studies to assess the knowledge and research capacity of supervisors to provide effective guidance 4. Examine how the academic and clinical workload of supervisors influences their ability to supervise students	[56]
7	Postgraduates and interns	Easternmost Medical College in India, 2015	1. Insufficient training 2. Limited funding 3. Lack of motivation 4. Absence of a mentorship program	1. Implement a well-designed competency-based curriculum 2. Establish a mentorship program 3. Provide incentives for engaging in research activities 4. Increase attention to training in research methodology	[57]
8	Medical residents	Three Joint Commission-accredited academic medical centers in the emirate of Abu Dhabi, 2017–2018	1. Limited time 2. Inadequate training in research methodology 3. Absence of a dedicated research budget	1. Application of the CREDIT-20 Survey to identify specific barriers faced by trainees 2. Dedicate structured-protected time for scholarly activities 3. Improve research methodology training 4. Enhance organizational support through the employing of research coordinators and statisticians 5. Establish a scholarly activity award system	[50]
9	Otolaryngology-Head & Neck Surgery residents below the age of 45	International Federation of Otolaryngological Societies meeting, 2017	1. Lack of dedicated time 2. Inadequate financial resources 3. Lack of research education	1. Provide dedicated research time 2. Balance clinical and research responsibilities by implementing structured research periods, such as half-days or monthly blocks 3. Seek funding opportunities and industry partnerships to alleviate insufficient resources 4. Enhance research education through formal training in methodologies, statistics, and grant writing via workshops and mentorship 5. Mentor residents and foster a supportive environment to alleviate the stress of balancing their duties	[58]
10	Medical students	Government Medical College, Nagpur, India, 2020	1. Limited time 2. Insufficient guidance 3. Inadequate funding	1. Organize a practical training course focused on research methodology 2. Establish journal clubs 3. Facilitate regular research presentations 4. Organize research workshops	[59]
11	Medical and dental students	King Khalid University in Saudi Arabia, 2020	1. Limitations in terms of time, skills, funding, and facilities 2. Restricted access to medical journals and related databases 3. Lack of recognition for research 4. Low attendance in research sessions 5. Hesitation to express thoughts	Intensive training and adequate support in research activities by organizing journal clubs, regular research presentations, training programs, and research workshops	[60]
12	Ophthalmology residents	Five residency programs within Saudi Arabia, 2020	1. Heavy workload associated with other educational tasks, including exams 2. Insufficient protected time for research 3. Abundance of regulations for obtaining ethical approval 4. Lack of interest 5. Inadequate proficiency in English	1. Provide protected time for research 2. Decrease the workload of other educational tasks 3. Facilitate the ethical approval process 4. Formalize mentorship programs and senior supervision 5. Incorporate formal instruction on manuscript writing, the basics of biostatistics, and research methodology in training program curricula	[61]
13	Medical students	Medical schools in six Arab countries, including Egypt, Algeria, Sudan, Jordan, Syria, and Palestine, 2020	1. Insufficient laboratory equipment 2. Prioritization of education over research 3. Lack of time due to educational responsibilities	1. Develop structured research skills programs that are evidence-based 2. Integrate research as a mandatory component of the medical curriculum 3. Provide dedicated time for research activities 4. Establish mentorship programs 5. Create a supportive environment that encourages research engagement 6. Enhance publication opportunities through initiatives that assist students in turning their research into publishable work, including workshops on writing and submission processes 7. Cultivate a positive research culture within medical schools to convert students’ favorable perceptions into active involvement in research 8. Encourage students to engage in practical research projects that are more likely to be publishable	[62]
14	Medical students	Medical schools from 26 countries, with a majority from Latin America, North America, and other regions, 2020	1. Lack of research opportunities 2. Lack of mentors 3. Lack of formal training 4. Challenges related to the COVID-19 pandemic	1. Enhance research training programs globally 2. Enhance initiatives linking medical school training to further career stages, such as residency and junior faculty levels 3. Implement programs that support research initiatives spanning different training environments 4. Adapt curricula to meet the constraints posed by the COVID-19 pandemic, potentially through online or standardized global approaches	[63]
15	Medical students	Government Medical College in Rajasthan, 2022	1. Insufficient time 2. Limited skills 3. Lake of cooperation between departments 4. Inadequate funding 5. Low motivation and interest	1. Implement a practical training course focused on research methodology 2. Establish research workshops and learning programs overseen by senior faculty members	[64]
16	Medical students	Al-Balqa Applied University, 2023	1. Insufficient training in medical research 2. Lack of research opportunities 3. Inadequate faculty support.	1. Integrate research into the curriculum 2. Design courses focused on research methodologies and critical thinking 3. Provide mentorship programs 4. Offer financial support for research projects 5. Foster a culture that values research by recognizing and rewarding faculty contributions	[65]

*Note* CREDIT-20: Clinical Research Excellence Development in Innovation and Technology. COVID-19: Coronavirus Disease 2019

#### Study 1

To examine the challenges faced by internal medicine residents in conducting scholarly work during their residency, a questionnaire was distributed to 138 residents who had presented their research at the 2002 annual session of the American College of Physicians-American Society of Internal Medicine [51].

#### Barriers

The most prevalent barriers reported by residents were insufficient time (79%), inadequate research skills (45%), and the absence of a research curriculum (44%). Despite facing challenges, most respondents found their scholarly project to be a valuable experience, and 69% of residents believed that research should be a mandatory component of residency programs [51].

#### Solutions suggestion

To enhance support for resident research in internal medicine residency programs, several recommendations can be considered: introducing and implementing a research curriculum that explicitly teaches research skills, providing protected time for residents to engage in scholarly work, establishing funding opportunities to support resident research endeavors, promoting mentorship and collaboration between residents and faculty members, and making research a mandatory component of residency training. These recommendations can potentially mitigate the barriers that residents face in conducting scholarly work, thereby increasing their chances of success in research activities [51].

#### Study 2

A study evaluated 13 medical programs across six Brazilian states by interviewing medical students to investigate the availability of scientific training programs and identify barriers to participation [52].

#### Barriers

The main barrier to participation was the lack of institutional incentives, which significantly hindered student involvement. Other barriers included inadequate infrastructure and limited mentorship time from professors [52].

#### Solutions suggestion

Medical schools should develop structured research programs to encourage student participation and implement policies providing incentives for students and faculty. The Brazilian government should prioritize enhancing medical student involvement in research by allocating resources for training opportunities. Research activities should be made a mandatory part of medical education, with curricula revised to ensure students acquire essential research skills. Collaboration with public and private sectors is also necessary to improve research facilities. While focused on Brazil, these findings are relevant to other developing countries facing similar challenges in medical education [52].

#### Study 3

Conducted at Kasturba Medical College, Mangalore, a study assessed students’ awareness of and practices related to research in the medical field. The findings revealed that many students involved in research expressed a willingness to consider it as a career, indicating that exposure to research can positively influence their career aspirations [53].

#### Barriers

The study identified several barriers to student involvement in research, including a shortage of time (52.1%), lack of financial or academic incentives (56.3%), and a general disinterest in pursuing research as a career (32.4%) [53].

#### Solutions suggestion

Medical schools should enhance research training, develop support systems, address perceptions of research careers, encourage student participation, and consider gender dynamics to foster a more research-oriented culture among medical students [53].

#### Study 4

A study aimed to examine how pediatric residency programs meet the Accreditation Council for Graduate Medical Education’s (ACGME) requirements for resident involvement in scholarly activities and to identify the characteristics of successful scholarly activity initiatives within these programs. Approximately 78.6% of pediatric residency programs required scholarly activity, with resident participation averaging 56% in original research, systematic reviews, and case reports. About half of the program directors expressed satisfaction with both resident participation and the quality of scholarly activity and research training provided. However, satisfaction levels were generally low, indicating potential areas for improvement in resident research training experiences. Successful programs emphasized teaching scientific inquiry and problem-solving skills and provided essential resources such as funding, research infrastructure, mentorship, and protected time to support resident scholarly activities [54].

#### Barriers

Program directors identified several obstacles to resident scholarly activity, including insufficient time, limited faculty expertise, and inadequate funding [54].

#### Solutions suggestion

Enhancing resident training through structured research programs can improve their knowledge and skills, positively influence career paths, and assist program directors in refining scholarly activity initiatives. Addressing variability in available resources, overcoming barriers, and addressing low satisfaction rates among program directors can help allocate resources more effectively, create a more supportive environment for research engagement, and ultimately enhance the quality of scholarly activities within pediatric residency programs. Additionally, implementing a scholarly activity requirement and providing faculty mentorship can significantly increase resident participation in research. Nevertheless, the need for standardized guidelines to ensure consistency and effectiveness in research training remains evident [54].

#### Study 5

A study conducted on medical students at Taibah University explored their perceptions, barriers, and practices related to medical research. The findings revealed that students generally held a favorable view of medical research, with this positive attitude being consistent across both male and female students. This indicates a shared recognition of the importance of research in the medical field. Notably, the practice of engaging in medical research was significantly higher among female students compared to their male counterparts [55].

#### Barriers

The research identified several barriers that hinder students from conducting and publishing their research. These barriers included inadequate research facilities, limited interest from faculty or mentors, and a lack of available samples or patients [55].

#### Solutions suggestion

The study highlights the need for improved research training in medical education to equip students with essential skills and confidence. It identifies barriers such as inadequate facilities and limited faculty engagement, suggesting that increased institutional support could enhance student participation. Notably, female students tend to engage more in research, indicating a need for initiatives to encourage greater male participation to achieve a better gender balance. Faculty mentorship is crucial, and training workshops can help faculty support students more effectively [55].

#### Study 6

An investigation highlighted the challenges faced by final-year medical students in Sudan and suggested implementing a small group learning model to improve research training and supervision. Most final-year medical students recognized the importance of research in the medical field and supported its inclusion in the medical curriculum. However, fewer students viewed research experience as a crucial requirement for residency training upon graduation. The primary motivation for pursuing a research-focused career was to enhance their professional standing as clinicians [56].

#### Barriers

The main factors deterring students from a research-focused career included the time-consuming nature of research and the belief that prioritizing clinical service was more significant than involvement in clinical research. The primary barriers to conducting research were insufficient funding, limited time, and the demands of the curriculum [56].

#### Solutions suggestion

Adopting a small group learning model is recommended to improve students’ research training and ensure effective supervision of group research projects. Implementing this model can lead to enhanced academic learning, better skill acquisition, increased student interest in research, reduced obstacles to student-led research, and a more efficient use of limited resources. To address gaps in research training, future efforts should focus on revising the curriculum and investigating student theses to identify deficiencies in supervision, skills, and knowledge. Additionally, further research is needed to evaluate the knowledge and research capacity of supervisors to ensure they can provide effective guidance. It is also important to examine how the academic and clinical workload of supervisors impacts their ability to supervise students effectively [56].

#### Study 7

Vairamani et al. studied the perceived barriers to research among medical students in India and found that, despite a positive attitude towards research, participants generally lacked sufficient knowledge. While students were interested in gaining more knowledge and actively participating in research, they faced several obstacles [57].

#### Barriers

The respondents identified insufficient training (87%), limited funding (76%), lack of motivation (67%), and the absence of a mentorship program (66%) as the primary reasons for their non-participation in research projects [57].

#### Solutions suggestion

Enhancing participation in research can be achieved through the implementation of a well-designed competency-based curriculum, the establishment of a mentorship program, and the provision of incentives for engaging in research activities. Additionally, it is important to emphasize training in research methodology, as it is a critical yet often neglected element in advancing medical science and healthcare [57].

#### Study 8

A study introduced the Clinical Research Excellence Development in Innovation and Technology (CREDIT-20) questionnaire to identify barriers to resident research participation in the emirate of Abu Dhabi. The data revealed a strong interest among residents in engaging in research. According to feedback from most trainees, 93% believed that research would enhance their critical thinking, while 92% acknowledged that it would significantly increase their medical knowledge [50].

#### Barriers

Limited time and inadequate training in research methodology emerged as the main challenges hindering research participation. Additionally, more than 50% of the residents identified the lack of a dedicated research budget as a significant organizational challenge within the program [50].

#### Solutions suggestion

The CREDIT-20 survey helps identify specific barriers faced by trainees and enables medical education leaders to implement targeted interventions. Strategies such as allocating structured and protected time for scholarly activities, improving training in research methodology, enhancing organizational support by employing research coordinators and statisticians, and implementing a scholarly activity award system could lead to increased research output at international academic medical centers [50].

#### Study 9

Fournier et al. identified research challenges faced by Otolaryngology-Head & Neck Surgery (OTL-HNS) residents under the age of 45 during the 2017 International Federation of Otolaryngological Societies (IFOS) meeting [58].

#### Barriers

Although research is crucial for generating valuable insights into patient care, most OTL-HNS residents face challenges in integrating research into their surgical curriculum. The primary barriers encountered by trainees include insufficient dedicated time (64%), inadequate financial resources (55%), and limited research education (45%). No significant differences were observed in these barriers across different countries [58].

#### Solutions suggestion

Residency programs should prioritize providing dedicated research time, as many residents view limited time as a significant barrier. Implementing structured research periods, such as half-days or monthly blocks, can help balance clinical and research responsibilities. Financial support is also crucial, as residents often face insufficient resources; programs should pursue funding opportunities and industry partnerships to alleviate this burden. Enhancing research education through formal training in methodologies, statistics, and grant writing via workshops and mentorship is essential. Additionally, faculty involvement is vital; faculty should mentor residents and create a supportive environment to ease the stress of balancing duties. Given that research barriers are similar globally, residency programs should tailor solutions to these challenges and promote international collaboration to improve research opportunities. Addressing these factors can lead to systemic changes that encourage greater research engagement among trainees [58].

#### Study 10

Sharma et al. examined the potential barriers faced by medical students at Government Medical College, Nagpur, India, when conducting research. Although many students had a positive attitude towards research, only a small percentage had actively conducted and published research [59].

#### Barriers

Limited time (75%), insufficient guidance (68%), and inadequate funding (67%) emerged as significant barriers impeding the research practice of medical students. Additionally, some students (39%) suggested incorporating research into the medical school curriculum, while 35% emphasized the importance of increasing awareness about research [59].

#### Solutions suggestion

To promote research practice, several measures are suggested. These include organizing practical training courses focused on research methodology, establishing journal clubs, facilitating regular research presentations, and conducting research workshops. These initiatives aim to provide medical students with the essential knowledge and skills needed for active involvement in scientific research [59].

#### Study 11

In a cross-sectional study conducted at King Khalid University in Saudi Arabia, the research obstacles experienced by medical and dental students were assessed. The study found that the majority of medical and dental students exhibited positive attitudes towards engaging in research [60].

#### Barriers

Medical and dental students faced several challenges, including time constraints, limited skills, insufficient funding and facilities, restricted access to medical journals and databases, lack of recognition for their research, low attendance at research sessions, and reluctance to voice their opinions [60].

#### Solutions suggestion

To equip medical students with the knowledge and skills essential for successfully conducting scientific research, this study recommended organizing journal clubs, regular research presentations, training programs, and research workshops [60].

#### Study 12

A study examined research challenges among ophthalmology residents across five residency programs in Saudi Arabia. The survey results indicated that a substantial majority of participants (80.3%) recognized the importance of research in advancing scientific and medical knowledge as well as contributing to education. Additionally, 78.9% of participants believed that research fosters the development of critical thinking skills, and 74.8% viewed research as a valuable asset when applying for fellowships [61].

#### Barriers

The most significant barrier to performing research was the heavy workload associated with other educational tasks, including exams. Other perceived barriers included insufficient protected time for research and the extensive regulations for obtaining ethical approval. The least frequently reported barriers were a lack of interest and inadequate proficiency in English [61].

#### Solutions suggestion

To address the challenges faced by ophthalmology residents in conducting research and to improve the integration of research into residency curricula, program directors should provide protected time for research, reduce the workload associated with other educational tasks, and streamline the ethical approval process. Additionally, formalizing mentorship programs and senior supervision is essential to help residents achieve their research goals. Training program curricula should also include formal instruction on manuscript writing, biostatistics, and research methodology. These measures are crucial for enabling residents to develop the skills necessary to produce high-quality research projects [61].

#### Study 13

Assar et al. evaluated the research barriers faced by medical students in six Arab countries. Most undergraduate medical students had a limited understanding of research but generally maintained a positive attitude towards its significance and practicality. Approximately one-third of students were involved in research projects, primarily focusing on cross-sectional studies and case reports, with an average of 0.5 publications per student [62].

#### Barriers

The key obstacles to research practice were inadequate laboratory equipment (68.1%), a focus on education rather than research (66.8%), and limited time due to educational commitments (66.1%) [62].

#### Solutions suggestion

To enhance research capabilities among medical students in the Arab world, it is crucial to develop structured, evidence-based research skills programs. Incorporating research as a mandatory part of the medical curriculum can help students balance their educational responsibilities while dedicating time to research. Establishing mentorship programs with experienced faculty can bridge knowledge gaps and facilitate successful outcomes. Institutions should address perceived barriers by creating a supportive environment that encourages research engagement and enhances publication opportunities. The low publication rates highlight the need for initiatives that assist students in turning research into publishable work, such as workshops on writing and submission processes. Cultivating a positive research culture within medical schools can transform students’ favorable perceptions into active involvement. Encouraging practical research projects that are more likely to be publishable can significantly improve students’ research experiences and outcomes [62].

#### Study 14

A study assessed medical students’ perceptions of research training globally and evaluated obstacles to conducting research during medical training. The study involved 318 medical students from 26 countries, with a majority from Latin America, North America, and other regions, providing a broad global perspective [63].

#### Barriers

Most students felt that research was crucial in medical training but reported a lack of support from their institutions. Common barriers included insufficient research opportunities, a lack of mentors, inadequate formal training, and challenges related to the Coronavirus Disease 2019 (COVID-19) pandemic [63].

#### Solutions suggestion

The findings underscore the need for enhanced research training programs globally, as well as initiatives that link medical school training to further career stages, such as residency and junior faculty levels. Additionally, implementing programs that support research initiatives across different training environments and adapting curricula to address constraints posed by the pandemic—potentially through online or standardized global approaches—is crucial [63].

#### Study 15

A study examined the research barriers experienced by medical students at Government Medical College in Rajasthan [64].

#### Barriers

Most medical students demonstrated a limited understanding of basic medical research. The primary barriers they faced in conducting research included insufficient time (81.8%), limited skills (81%), lack of cooperation between departments (78.5%), inadequate funding (76.3%), and low motivation (74.4%) and interest (73.4%) [64].

#### Solutions suggestion

Improving students’ research skills can be achieved by introducing practical training courses in research methodology and setting up research workshops and learning programs guided by senior faculty members [64].

#### Study 16

A cross-sectional survey of medical students at Al-Balqa Applied University indicated that their attitude toward research was generally positive from the second year onward, suggesting that early exposure to research concepts fosters a favorable outlook. However, students exhibited limited knowledge of research processes. The study found that knowledge significantly increased with academic progression, with each additional year correlating to a 0.25 rise in knowledge scores, highlighting the importance of advancing education [65].

#### Barriers

The key challenges to participating in research were insufficient training in medical research, limited opportunities, and inadequate faculty support [65].

#### Solutions suggestion

To enhance research involvement, educational policymakers and medical educators should integrate research into the curriculum, create courses on research methodologies and critical thinking, offer mentorship programs, and provide financial support for research projects. Additionally, fostering a culture that values research by recognizing and rewarding faculty contributions is essential [65].

### Boosting research engagement in medical education: effective programs and mentorship strategies

Despite the barriers identified in previous studies, several effective strategies have emerged to enhance research engagement and productivity among undergraduate medical students and residents through structured programs and mentorship. A notable intervention is the Resident Research Mentoring Team (RRMT) program, which has significantly increased the likelihood of residents publishing and presenting their research projects. It has been demonstrated that residents participating in the RRMT program exhibited improved research productivity compared to those who did not participate, and this effect persisted even after controlling for various factors. This underscores the robust impact of dedicated mentorship. The RRMT, composed of faculty, statisticians, and research staff, provided essential guidance, thereby enhancing residents’ research skills and output. These findings highlight the importance of investing in mentorship and structured research support to boost scholarly activity and contribute to medical advancements [66]. Similarly, a structured research program implemented at the University of Colorado’s obstetrics and gynecology residency exemplifies effective strategies for enhancing research productivity. This program introduced a formal research curriculum, summer didactic sessions, and internal grant funding, which collectively improved scholarly output among residents. Key factors contributing to this success included the active involvement of dedicated faculty as Assistant Residency Program Directors (APDs) and ongoing administrative support. However, the study also identified challenges such as mentor burnout and a lack of mentorship expertise, which can hinder the sustainability of such initiatives. Addressing these barriers is essential to maintain and enhance the positive impact of structured research programs on residents’ career prospects, particularly in academic settings [67]. The Student Research Forum (SRF) at a private medical school in Pakistan exemplifies an innovative approach to fostering research skills among medical students. This student-led initiative effectively cultivated mentor-mentee relationships and promoted a culture of academic research. By leveraging social media and organizing Journal Clubs, the SRF provided students with insights into recent advancements and research opportunities, which led to increased publications. The practical strategies employed, including mentorship sessions and workshops, proved effective even amid challenges like the COVID-19 pandemic. Continuous evaluation of these initiatives is crucial to ensure their effectiveness and sustainability in building research capacity and enhancing student engagement [68]. Finally, a study examining curriculum initiatives for incorporating research into medical education highlighted the need for flexibility based on a medical school’s resources, curriculum, and student needs. It stressed the importance of addressing factors such as cost, mentor availability, and student motivation when implementing research initiatives. The findings suggest that tailored approaches can significantly enhance research experiences for medical students, ultimately preparing them for future academic and clinical challenges [69]. To sum up, these studies collectively illustrate that effective mentorship, structured programs, and flexible curriculum initiatives are pivotal for enhancing research engagement and productivity in medical education. By fostering a supportive research environment, medical institutions can advance scholarly activity and improve the overall quality of medical training and patient care.

## Conclusions

Exposure to research is essential for undergraduate medical students and residents, as it fosters a comprehensive understanding of their field and equips them with the skills necessary for successful careers in medicine. Research experience not only enhances opportunities for pursuing physician-scientist professions but also plays a critical role in shaping well-rounded medical professionals. However, fostering positive attitudes toward scientific research is crucial, as negative attitudes can diminish curiosity and hinder engagement in research activities or careers. The integrity of research practices in residency selection is another vital consideration. Issues such as the arms race, misrepresentation, and the balance between quality and quantity can undermine the credibility of the selection process. Additionally, addressing the challenge of balancing research and clinical training is essential, particularly in promoting equity and inclusion among all medical trainees. Despite the benefits of research, many students and residents face significant challenges that can adversely affect both the quantity and quality of research output, impacting the broader medical field. To address the serious shortage of physician-researchers and enhance evidence-based medical practice, it is imperative for medical schools to prioritize research education and create opportunities for engagement. This includes developing effective programs and mentorship strategies that encourage participation in research, while also addressing socioeconomic and cultural issues, curriculum-related concerns, and the need for skill development. By fostering a supportive research environment and motivating students and residents to engage in research activities, we can cultivate a generation of medical professionals who are not only skilled clinicians but also innovative researchers dedicated to advancing the field of medicine.

### Lessons for practice

- Incorporating structured research programs is vital for equipping undergraduate medical students and residents with the necessary resources, mentorship, and training to succeed in their research efforts. These programs provide a supportive framework that encourages active research engagement and development.
- Establishing protected research time within medical education and residency is essential for allowing students and residents to concentrate on their research without the pressures of clinical duties. This dedicated time is crucial for producing meaningful research contributions and advancing their scholarly pursuits.
- Encouraging collaboration and resource sharing among undergraduate medical students, residents, and experienced researchers can greatly enhance the effectiveness and outcomes of research projects. Such partnerships not only optimize the use of available resources but also build a collaborative academic community.
- Choosing research topics that align with students’ and residents’ personal interests and passions can significantly boost motivation and engagement. When individuals are passionate about their research, they are more likely to produce impactful and innovative work.
- Finally, setting realistic research goals within the limits of available time and resources is key to improving both the quantity and quality of research output. Achievable goals help maintain motivation and ensure steady progress, leading to more effective and rewarding research experiences.

## Data Availability

All data generated or analyzed during this study are included in this published article.

## Abbreviations

ACGME: Accreditation Council for Graduate Medical Education’s
APDs: Assistant Residency Program Directors
COVID-19: Coronavirus Disease 2019
CREDIT-20: Clinical Research Excellence Development in Innovation and Technology
IFOS: International Federation of Otolaryngological Societies
MeSH: Medical Subject Headings
MSTP: Medical Scientist Training Program
OTL-HNS: Otolaryngology-Head & Neck Surgery
RRMT: Resident Research Mentoring Team
SRF: Student Research Forum
URIM: Underrepresented in Medicine
USMLE: United States Medical Licensing Examination

## References

[1] Halalau A, Holmes B, Rogers-Snyr A, Donisan T, Nielsen E, Cerqueira TL, Guyatt G. Evidence-based medicine curricula and barriers for physicians in training: a scoping review. Int J Med Educ. 2021;12:101–24. 34053914 10.5116/ijme.6097.ccc0 PMC8411338

[2] Rosenkranz SK, Wang S, Hu W. Motivating medical students to do research: a mixed methods study using self-determination theory. BMC Med Educ. 2015;15(1):1–13. 26032008 10.1186/s12909-015-0379-1 PMC4486085

[3] Ommering BW, van Blankenstein FM, Waaijer CJ, Dekker FW. Future physician-scientists: could we catch them young? Factors influencing intrinsic and extrinsic motivation for research among first-year medical students. Perspect Med Educ. 2018;7:248–55. 30006870 10.1007/s40037-018-0440-y PMC6086821

[4] Utz PJ, Jain MK, Cheung VG, Kobilka BK, Lefkowitz R, Yamada T, Dzau VJ. Translating science to medicine: the case for physician-scientists. Am Assoc Adv Sci; 2022. p. eabg7852. 10.1126/scitranslmed.abg7852 35171650

[5] Jain MK, Cheung VG, Utz PJ, Kobilka BK, Yamada T, Lefkowitz R. Saving the endangered physician-scientist—a plan for accelerating medical breakthroughs. N Engl J Med. 2019;381(5):399–402. 31365796 10.1056/NEJMp1904482

[6] Rahman S, Majumder MAA, Shaban SF, Rahman N, Ahmed M, Abdulrahman KB, D’souza UJ. Physician participation in clinical research and trials: issues and approaches. Adv Med Educ Pract. 2011:85–93. 10.2147/AMEP.S14103 PMC3661249 23745079

[7] Pathipati AS, Taleghani N, Taleghani NN. Research in medical school: a survey evaluating why medical students take research years. Cureus. 2016;8(8):e741. 27672532 10.7759/cureus.741 PMC5026499

[8] Mitwalli H, Al Ghamdi K, Moussa N. Perceptions, attitudes, and practices towards research among resident physicians in training in Saudi Arabia. East Mediterr Health J. 2014;20(2):99–104. 24945558

[9] Chang Y, Ramnanan CJ. A review of literature on medical students and scholarly research: experiences, attitudes, and outcomes. Acad Med. 2015;90(8):1162–73. 25853690 10.1097/ACM.0000000000000702

[10] Barzkar F, Baradaran HR, Koohpayehzadeh J. Knowledge, attitudes and practice of physicians toward evidence-based medicine: a systematic review. J Evid Based Med. 2018;11(4):246–51. 30430759 10.1111/jebm.12325

[11] Gharaibeh A, Mousa YS. Should research thesis be a prerequisite for doctor of medicine degree? a cross-sectional study at Jordan university of science and technology. Int J Med Stud. 2014;2(1):8–12.

[12] O’Brien J, D’Eon M. Re-thinking clinical research training in residency. Can Med Educ J. 2014;5(1):e58. 26451223 PMC4563610

[13] Andriole DA, Klingensmith ME, Fields RC, Jeffe DB. Is dedicated research time during surgery residency associated with surgeons’ future career paths? A national study. Ann Surg. 2020;271(3):590–97. 30829693 10.1097/SLA.0000000000003015 PMC6401322

[14] Seaburg LA, Wang AT, West CP, Reed DA, Halvorsen AJ, Engstler G, Oxentenko AS, Beckman TJ. Associations between resident physicians’ publications and clinical performance during residency training. BMC Med Educ. 2016;16(1):1–6. 26786879 10.1186/s12909-016-0543-2 PMC4717564

[15] Luz, PLd. Physician-Researcher, Medical Practice and Research: the importance of the physician-researcher in current medicine. Arq Bras Cardiol. 2022;119:801–3. 36453772 10.36660/abc.20220099 PMC9750216

[16] Hernandez-Suarez DF, San Martin MT, Colon-Marquez JA, Jimenez-Velazquez IZ, Lopez-Candales A. Continued need for clinical research in medical residency: from physician to clinical-translational researcher. P R Health Sci J. 2017;36(1):44–6. 28266700 PMC5536838

[17] Sadaba JR, Urso S. Does the introduction of duty-hour restriction in the United States negatively affect the operative volume of surgical trainees? Interact Cardiovasc Thorac Surg. 2011;13(3):316–9. 21719511 10.1510/icvts.2011.270363

[18] Bilal M, Haseeb A, Mari A, Ahmed S, Khan MAS, Saad M. Knowledge, attitudes, and barriers toward research among medical students of Karachi. Cureus. 2019;11(9):e5599. 31700712 10.7759/cureus.5599 PMC6822902

[19] Truong HT, Chan JJ, Leong WL, Sultana R, Koh DL, Sng BL. Interest and experience of anaesthesiology residents in doing research during residency training. Indian J Anaesth. 2019;63(1):42–8. 30745612 10.4103/ija.IJA_543_18 PMC6341890

[20] Pallamparthy S, Basavareddy A. Knowledge, attitude, practice, and barriers toward research among medical students: a cross-sectional questionnaire-based survey. Perspect Clin Res. 2019;10(2):73–8. 31008073 10.4103/picr.PICR_1_18 PMC6463502

[21] Chellaiyan VG, Manoharan A, Jasmine M, Liaquathali F. Medical research: perception and barriers to its practice among medical school students of Chennai. J Educ Health Promot. 2019;8:134. 31463319 10.4103/jehp.jehp_464_18 PMC6691744

[22] Shah SMM, Sohail M, Ahmad KM, Imtiaz F, Iftikhar S. Grooming future physician-scientists: evaluating the impact of research motivations, practices, and perceived barriers towards the uptake of an academic career among medical students. Cureus. 2017;9(12):e1991. 29503785 10.7759/cureus.1991 PMC5828671

[23] Furuya H, Brenner D, Rosser CJ. On the brink of extinction: the future of translational physician-scientists in the United States. J Transl Med. 2017;15(1):1–3. 28460639 10.1186/s12967-017-1188-6 PMC5412034

[24] Funston G, Piper RJ, Connell C, Foden P, Young AM, O’Neill P. Medical student perceptions of research and research-orientated careers: an international questionnaire study. Med Teach. 2016;38(10):1041–8. 27008336 10.3109/0142159X.2016.1150981

[25] AO CB. Why all medical students need to experience research. Aust Med Stud J. 2016;34:10.

[26] DeFranco DB, Sowa G. The importance of basic science and research training for the next generation of physicians and physician scientists. Oxford University Press; 2014. pp. 1919–21. 10.1210/me.2014-1343 PMC5414780 25437019

[27] Al-Busaidi IS, Wells CI, Wilkinson TJ. Publication in a medical student journal predicts short-and long-term academic success: a matched-cohort study. BMC Med Educ. 2019;19:1–7. 31324236 10.1186/s12909-019-1704-x PMC6642564

[28] Solomon SS, Tom SC, Pichert J, Wasserman D, Powers AC. Impact of medical student research in the development of physician-scientists. J Investig Med. 2003;51(3):149–56. 10.1136/jim-51-03-17 12769197

[29] Elliott B, Carmody JB. Publish or perish: the research arms race in residency selection. J Grad Med Educ. 2023;15(5):524–7. 37781419 10.4300/JGME-D-23-00262.1 PMC10539143

[30] Wolfson RK, Fairchild PC, Bahner I, Baxa DM, Birnbaum DR, Chaudhry SI, Chretien KC, DeFranco DB, Deptola AZ, LaConte LE. Residency program directors’ views on research conducted during medical school: a national survey. Acad Med. 2023;98(10):1185–95. 37099328 10.1097/ACM.0000000000005256 PMC10516175

[31] Radulovich NP, Burke S, Brown NJ, Jones B, Antongiovanni J, Nanu D, Roll J. The importance of research experience with a scoreless step 1: a student survey at a community-based medical school. Cureus. 2023;15(8):e43476. 37711915 10.7759/cureus.43476 PMC10499365

[32] Kistka HM, Nayeri A, Wang L, Dow J, Chandrasekhar R, Chambless LB. Publication misrepresentation among neurosurgery residency applicants: an increasing problem. J Neurosurg. 2016;124(1):193–8. 26207605 10.3171/2014.12.JNS141990

[33] Karri PV, Tahseen D, Gupta R, Grant-Kels JM, Narala S, Patel AB. Can quality keep up with quantity—longitudinal trends in research output for the dermatology residency match. Clin Dermatol. 2021;39(6):1039–45. 34920822 10.1016/j.clindermatol.2021.07.007

[34] Cerasani M, Omoruan M, Rieber C, Nguyen M, Mason HR, Clair B, Stain SC, Mason AR, Levin LS. Demographic Factors and Medical School Experiences Associated with Students’ intention to pursue orthopaedic surgery and practice in Underserved Areas. JBJS Open Access. 2023;8(1):e22. 10.2106/JBJS.OA.22.00016 PMC9851675 36698985

[35] Nguyen M, Chaudhry SI, Asabor E, Desai MM, Lett E, Cavazos JE, Mason HR, Boatright D. Variation in research experiences and publications during medical school by sex and race and ethnicity. JAMA Netw Open. 2022;5(10):e2238520–2238520. 36282497 10.1001/jamanetworkopen.2022.38520 PMC9597391

[36] Klowak J, Elsharawi R, Whyte R, Costa A, Riva J. Predictors of medical student interest and confidence in research during medical school. Can Med Educ J. 2018;9(3):e4. 30140343 PMC6104316

[37] Meraj L, Gul N, Zubaidazain IA, Iram F, Khan AS. Perceptions and attitudes towards research amongst medical students at Shifa College of Medicine. JPMA. 2016;66(2):165–9. 26819161

[38] Hames K, Patlas M, Duszak R. Barriers to resident research in radiology: a Canadian perspective. Can Assoc Radiol J. 2018;69(3):260–5. 30078398 10.1016/j.carj.2018.03.006

[39] Mansi A, Karam WN, Chaaban MR. Attitudes of residents and program directors towards research in otolaryngology residency. Ann Otol Rhinol Laryngol. 2019;128(1):28–35. 30371115 10.1177/0003489418804565

[40] Silcox LC, Ashbury TL, VanDenKerkhof EG, Milne B. Residents’ and program directors’ attitudes toward research during anesthesiology training: a Canadian perspective. Anesth Analg. 2006;102(3):859–64. 16492841 10.1213/01.ane.0000194874.28870.fd

[41] Siemens DR, Punnen S, Wong J, Kanji N. A survey on the attitudes towards research in medical school. BMC Med Educ. 2010;10(1):1–7. 20096112 10.1186/1472-6920-10-4 PMC2823602

[42] Clancy AA, Posner G. Attitudes toward research during residency: a survey of Canadian residents in obstetrics and gynecology. J Surg Educ. 2015;72(5):836–43. 25921189 10.1016/j.jsurg.2015.02.007

[43] Jaroonvanichkul V, Deerojanawong J. Residents’ obstacles and attitudes toward Research during Residency Training. J Med Assoc Thai. 2016;99(2):239–44. 27249906

[44] Chan JY, Narasimhalu K, Goh O, Xin X, Wong TY, Thumboo J, Phua GC. Resident research: why some do and others don’t. Singap Med J. 2017;58(4):212–17. 10.11622/smedj.2016059 PMC5392607 26976220

[45] Al-Taha M, Al Youha S, Al-halabi B, Stone J, Retrouvey H, Samargandi O, Efanov JI, Stein M, Morzycki A, Augustine H. Barriers and attitudes to research among residents in plastic and reconstructive surgery: a national multicenter cross-sectional study. J Surg Educ. 2017;74(6):1094–104. 28551364 10.1016/j.jsurg.2017.04.004

[46] Habineza H, Nsanzabaganwa C, Nyirimanzi N, Umuhoza C, Cartledge K, Conard C, Cartledge P. Perceived attitudes of the importance and barriers to research amongst Rwandan interns and pediatric residents–a cross-sectional study. BMC Med Educ. 2019;19(1):1–8. 30606184 10.1186/s12909-018-1425-6 PMC6318911

[47] Pop AI, Lotrean LM, Buzoianu AD, Suciu SM, Florea M. Attitudes and practices regarding Research among Romanian Medical Undergraduate Students. Int J Environ Res Public Health. 2022;19(3):1872. 35162894 10.3390/ijerph19031872 PMC8835173

[48] Cogswell PM, Deitte LA, Donnelly EF, Morgan VL, Omary RA. Attitudes of radiology program directors toward MD-PhD trainees, resident research productivity, and dedicated research time. Acad Radiol. 2018;25(6):733–8. 29530487 10.1016/j.acra.2018.01.029

[49] Riva JJ, Elsharawi R, Daza J, Toma A, Whyte R, Agarwal G, Busse JW. Medical students’ challenges and suggestions regarding research training: a synthesis of comments from a cross-sectional survey. Can Med Educ J. 2019;10(3):e91. 31388382 PMC6681923

[50] Nair SC, Ibrahim H, Almarzoqi F, Alkhemeiri A, Sreedharan J. Addressing research barriers and facilitators in medical residency. J Fam Med Prim Care. 2019;8(3):1145–50. 10.4103/jfmpc.jfmpc_38_19 PMC6482775 31041265

[51] Rivera JA, Levine RB, Wright SM. Completing a scholarly project during residency training: perspectives of residents who have been successful. J Gen Intern Med. 2005;20(4):366–9. 15857496 10.1111/j.1525-1497.2005.04157.x PMC1490090

[52] de Oliveira NA, Luz MR, Saraiva RM, Alves LA. Student views of research training programmes in medical schools. Med Educ. 2011;45(7):748–55. 21649708 10.1111/j.1365-2923.2011.03986.x

[53] Kumar HH, Jayaram S, Kumar GS, Vinita J, Rohit S, Satish M, Shusruth K. Perception, practices towards research and predictors of research career among UG medical students from coastal South India: a cross-sectional study. Indian J Community Med. 2009;34(4):306–9. 20165623 10.4103/0970-0218.58388 PMC2822190

[54] Abramson EL, Naifeh MM, Stevenson MD, Todd C, Henry ED, Chiu Y-L, Gerber LM, Li S-TT. Research training among pediatric residency programs: a national assessment. Acad Med. 2014;89(12):1674–80. 25006705 10.1097/ACM.0000000000000404 PMC4315313

[55] Noorelahi MM, Soubhanneyaz AA, Kasim KA. Perceptions, barriers, and practices of medical research among students at Taibah College of Medicine, Madinah, Saudi Arabia. Adv Med Educ Pract. 2015;479–85. 10.2147/AMEP.S83978 PMC4500619 26185479

[56] Osman T. Medical students’ perceptions towards research at a Sudanese University. BMC Med Educ. 2016;16:1–6. 27682259 10.1186/s12909-016-0776-0 PMC5041407

[57] Vairamani CR, Akoijam BS. Knowledge, attitude and perceived barriers towards conducting research among students in a medical college, India. Int J Community Med Public Health. 2018;5(2):806–10.

[58] Fournier I, Stephenson K, Fakhry N, Jia H, Sampathkumar R, Lechien J, Melkane A, Bahgat A, Lopes KDC, Kennel T. Barriers to research among residents in Otolaryngology-Head & Neck surgery around the world. Eur Ann Otorhinolaryngol Head Neck Dis. 2019;136(3):S3–7. 30143399 10.1016/j.anorl.2018.06.006

[59] Sharma SK, Thatikonda N, Ukey UU, Knowledge. Attitude, Practice and Barriers for Research amongst medical students of GMC, Nagpur. J Res Med Dent Sci. 2021;9(4):41–7.

[60] Alsaleem SA, Alkhairi MAY, Alzahrani MAA, Alwadai MI, Alqahtani SSA, Alaseri YFY, Alqarni MAM, Assiri SA, Alsaleem MA, Mahmood SE. Challenges and barriers toward medical research among medical and dental students at King Khalid University, Abha, Kingdom of Saudi Arabia. Front Public Health. 2021;9:706778. 34490190 10.3389/fpubh.2021.706778 PMC8417604

[61] Al Saeed AA, AlEnezi SH, Aljindan M, Alwadani F, Al Owaifeer AM. Experience, attitude, and perceived barriers toward Research among Ophthalmology residents in Saudi Arabia: A National Cross-sectional Study. Clin Ophthalmol. 2022:265–72. 10.2147/OPTH.S348647 PMC8820453 35140456

[62] Assar A, Matar SG, Hasabo EA, Elsayed SM, Zaazouee MS, Hamdallah A, Elshanbary AA, Khaled A, Badr H, Abukmail H. Knowledge, attitudes, practices and perceived barriers towards research in undergraduate medical students of six arab countries. BMC Med Educ. 2022;22(1):1–11. 35042492 10.1186/s12909-022-03121-3 PMC8767733

[63] Navarro SM, Stewart K, Tessier KM, Berhane A, Alvarado SP, Tafirei T, Abdi H, Keil EJ, Tuttle T, Rickard J. Medical students’ perceptions of clinical and Research Training: An International needs Assessment of 26 countries. Int J Transl Med Res Public Health. 2023;7(2):e454. 37854359 10.21106/ijtmrph.454 PMC10583816

[64] Sayed MA, Mudgal S, Sharma T, Khan C, Juyal D, Gupta G. Knowledge, attitude, practices and perceived barriers towards research among the undergraduate medical students of Government Medical College in Rajasthan. J Pharm Sci Res. 2022;14(5):739–45.

[65] Abusamak M, AlQato S, Alrfooh HH, Altheeb R, Bazbaz L, Suleiman R, Almansi A, Karajeh A, Alkhalaileh A, Al-Amer R. Knowledge, attitudes, practices and barriers of medical research among undergraduate medical students in Jordan: a cross-sectional survey. BMC Med Educ. 2024;24(1):23. 38178119 10.1186/s12909-023-05002-9 PMC10768081

[66] Haas DM, Hadaie B, Ramirez M, Shanks AL, Scott NP. Resident Research Mentoring teams: a support program to increase Resident Research Productivity. J Grad Med Educ. 2023;15(3):365–72. 37363673 10.4300/JGME-D-22-00499.1 PMC10286925

[67] Guiahi M, Mazzoni S, Metz T, Alston M. Impact of a structured obstetrics and gynecology residency research program. Am J Obstet Gynecol. 2019;221(4):364–5. 31279443 10.1016/j.ajog.2019.05.047

[68] Haroon MA, Noorali AA, Khan AS, Hussain MH, Advani R, Sami A, Merchant AA, Khan AA, Baloch SG, Tharwani A. Implementation evaluation of a medical student-led intervention to enhance students’ engagement with research: findings and lessons learned. PLoS ONE. 2023;18(8):e0290867. 37651371 10.1371/journal.pone.0290867 PMC10470873

[69] Carberry C, McCombe G, Tobin H, Stokes D, Last J, Bury G, Cullen W. Curriculum initiatives to enhance research skills acquisition by medical students: a scoping review. BMC Med Educ. 2021;21(1):312. 34078364 10.1186/s12909-021-02754-0 PMC8173745

